# The Constructor: a web application optimizing cloning strategies based on modules from the registry of standard biological parts

**DOI:** 10.1186/1754-1611-6-14

**Published:** 2012-09-04

**Authors:** Matthijn C Hesselman, Jasper J Koehorst, Thijs Slijkhuis, Dorett I Odoni, Floor Hugenholtz, Mark W J van Passel

**Affiliations:** 1 iGEM Wageningen UR, Wageningen University, Wageningen, The Netherlands; 2 Laboratory of Microbiology, Wageningen University, Wageningen, The Netherlands; 3 Laboratory of Systems and Synthetic Biology, Wageningen University, Wageningen, The Netherlands

**Keywords:** Synthetic Biology, BioBrick parts, Transcription units, Genetic circuit

## Abstract

Synthetic biology is an emerging field that combines molecular biology with engineering principles, which requires abstraction levels applied to a modular biological componentry. The Registry of Standard Biological Parts harbours such a repository of standardized parts, and thereby facilitates the combination of complex molecular modules to novel genetic circuits and devices. However, since finding the best parts for a pre-determined genetic design can be time consuming, we devised the Constructor, a web tool that recommends the smallest number of cloning steps for pre-designed circuits, and implements user-defined quality checks.

We present the Constructor ( http://www.systemsbiology.nl/the_constructor) as a constructive web tool that simplifies the *in silico* assembly of pre-designed gene circuitries from standard parts, reducing both planning and subsequent cloning time.

## Biological engineering

In recent years striking biological circuits have been fabricated, often resembling devices from electrical engineering. Examples include a genetic toggle switch [1], oscillators [2], a rewritable memory unit [3], but also more complex features such as a DNA-guided assembly line [4]. These sorts of devices provide a starting ground for further engineering. However, next to the development of stable and streamlined microbial chassis [5-7], biological engineering requires accessible, modular, standard parts with reliable characteristics. Even though several repositories have been constructed [8], the most well-known library is the Registry of Standard Biological Parts ( http://partsregistry.org). It is widely used in the international Genetically Engineered Machine (iGEM) competition [9] aimed at teaching synthetic biology to undergraduate students [10], [11], but also outside of this competition [12]. Users can design new devices from so-called BioBrick parts, the standard genetic parts, and are encouraged to submit new parts and devices to this Registry, which has grown to approximately 20,000 entries in 2012.

However, the vast number of entries in this Registry as well as the numerous parts with an unconfirmed status can frustrate the straightforward design of new genetic circuits. Furthermore, often several variants of parts and devices are available in the databases, which makes it difficult to find the shortest and most reliable strategy to clone new gene circuits. We found a need to automate querying the Registry in order to find the most straightforward cloning strategy for any pre-designed genetic circuit. This would simultaneously reduce time spent on finding appropriate parts in the Registry. We therefore set out to code the Constructor, a web-based application that recommends the smallest number of biological parts for a user-defined gene circuit, reducing both dry-lab and wet-lab time.

## The Constructor

**Figure 1:**
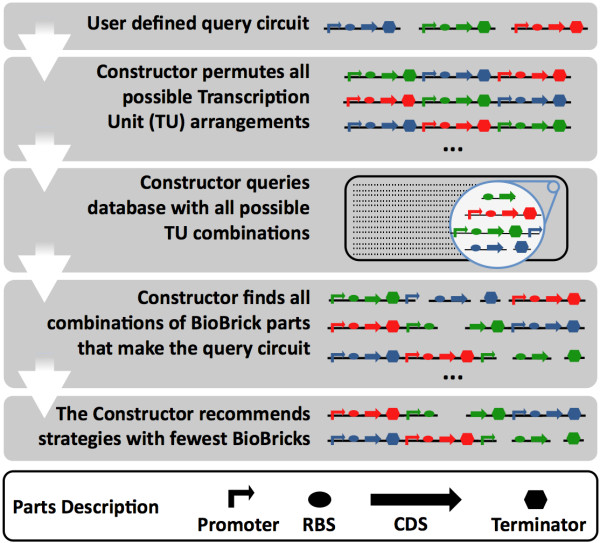
**Schematic representation of the functionality of the Constructor.** The user fills the codes of the parts of the query circuit (based on the vocabulary of the Registry). Next, the Constructor finds all arrangements of the Transcription Units (TUs). The functionality of the gene circuit is supposed to be independent of the order of the different TUs, and the Constructor tries to build all possible arrangements from all BioBrick parts in the Registry. Finally, the Constructor recommends up to three sets of BioBrick parts with which the genetic circuit can be made, based on the smallest number of parts, aiming for the fewest cloning steps. The symbols of the various parts (promoter, ribosomal binding site (RBS), coding sequence (CDS) and terminator) are explained in the lower panel.

The web interface of the Constructor accepts user-defined genetic circuits built from individual transcription units (TUs). A TU consists of a promoter, a ribosomal binding site (RBS), a coding sequence (CDS) and a terminator (see also Figure 1). Arrangements with multiple RBSs and CDSs between a promoter and terminator, i.e., an operon, are allowed. Next, users can give a complex genetic circuit consisting of a number of TUs as input. To our knowledge, there is no evidence that the physical location of TUs on a plasmid has any relevance for the function of the genetic circuit. Therefore the Constructor permutes all possible arrangements of the different TUs making up a circuit. For example, if the genetic circuit consists of three separate TUs (in the order A-B-C, where each letter signifies a TU), the Constructor uses all six possible arrangements in its query (A-B-C, B-A-C, B-C-A,…).

For each circuit arrangement, an SQL database containing information of ~20,000 parts (assembly standard 10 compatible) is queried. Since each circuit arrangement has a different sequence of genetic elements, each arrangement could collect a few arrangement-specific BioBrick parts. Through the combination of these specific BioBrick parts and the unique arrangement of genetic elements, some circuit arrangements could be constructed with fewer parts than others. An extra filter can be set if required on the availability, validity and quality of selected BioBrick parts by The Constructor.

Of all these possible combinations, the top three arrangements that could be assembled with the smallest number of BioBrick parts are shown as a recommendation to the user (Figure 1).

## Validation

We tested several proposed cloning strategies from five iGEM 2011 team projects, and compared these to the cloning strategies recommended by the Constructor. We find that the number of cloning steps predicted by the Constructor with appropriate parts criteria is generally smaller than those that were undertaken by the teams (Table 1), indicating the usefulness of the Constructor in optimizing cloning strategies. Specifically, in four out of five cases, the Constructor recommends cloning strategies that involve fewer BioBrick parts, and therefore fewer cloning steps. Also, in most cases, the processing time of the Constructor is less than two minutes. The most complex genetic circuit, from the iGEM team UANL_Mty-Mexico, consisting of seven transcriptional units, took approximately ten hours to run. Still, this is expected to be less than manually querying the Registry for the optimal cloning strategy.

**Table 1:** Validation of the Constructor by comparing five 2011 iGEM team cloning approaches to the recommendation by the Constructor

**iGEM 2011 team**	**iGEM project title**	**# of TUs in the designed circuit**	**# of used Biobrick Parts**		**Processing time (min)**
			**Used by team**	**Recommended by the Constructor**	
KAIST-Korea^1^	*E. casso*	2	11	7	< 1 min
UANL_Mty-Mexico^2^	S.C.I.E.N.C.E. : Simple Code Interpretation Enabling Circuit in *E. coli*	7	26	25	674 min
XMU-China^3^	i-*ccdB*: intelligent Control of Cell Density in Bacteria	3	6	6	< 1 min
NTNU-Trondheim^4^	Red Fluorescent Stress Sensor	2	5	4	< 1 min
Wageningen UR^5^	The Synchroscillator: a Synchronized Oscillatory System	4	7	6	< 2 min

^1^http://2011.igem.org/Team:KAIST-Korea.

^2^http://2011.igem.org/Team:UANL_Mty-Mexico.

^3^http://2011.igem.org/Team:XMU-China.

^4^http://2011.igem.org/Team:NTNU_Trondheim.

^5^http://2011.igem.org/Team:Wageningen_UR.

In these examples, the gene circuits contained between 2 and 7 Transcription Units (TUs).

## Potential extensions

The Constructor facilitates the cloning strategy of complex pre-designed genetic circuits from elements of the Registry of Standard Biological Parts. Although the Constructor specifically focuses on this Registry, the same search algorithms for assembly optimization recommendations can be applied to other extensiveand well-defined parts libraries. Furthermore, theConstructor uses a straightforward transcriptional unit concept, which could be expanded by including different parts such as splicing signals. Finally, the web tool could be further optimised by suggesting alternatives for certain parts, like available reporter genes with another fluorescent ability, or different inducible promoters.

## Availability

The Constructor is available at http://www.systemsbiology.nl/the_Constructor. Help functions and a tutorial are provided with test cases of user defined gene circuits. All scripts are available from the authors upon request.

## Competing interest

The author declares that he has no competing interests.

## Authors’ contributions

MCH conceived the initial design of the software, programmed the algorithms, and helped draft the manuscript. JJK participated in the design of the study, further developed the algorithms, added functionalities and helped to draft the manuscript. TS participated in the design of the application and added functionalities. DIO participated in the initial design of the application. FH participated in the design and coordination of the study and helped to draft the manuscript. MWJvP participated in the design and coordination of the study, added functionalities, and helped to draft the manuscript. All authors read and approved the final manuscript.

## References

[B1] Gardner TS Cantor CR Collins JJ Construction of a genetic toggle switch in Escherichia coli Nature 2000 403 6767 339 342 10.1038/35002131 10659857

[B2] Elowitz MB Leibler S A synthetic oscillatory network of transcriptional regulators Nature 2000 403 6767 335 338 10.1038/35002125 10659856

[B3] Bonnet J Subsoontorn P Endy D Rewritable digital data storage in live cells via engineered control of recombination directionality Proc Natl Acad Sci USA 2012 109 23 8884 8889 10.1073/pnas.1202344109 22615351 PMC3384180

[B4] Conrado RJ Wu GC Boock JT Xu H Chen SY Lebar T Turnsek J Tomsic N Avbelj M Gaber R DNA-guided assembly of biosynthetic pathways promotes improved catalytic efficiency Nucleic Acids Res 2011 40 4 1879 1889 22021385 10.1093/nar/gkr888 PMC3287197

[B5] Leprince A van Passel MW Dos Santos VA Streamlining genomes: toward the generation of simplified and stabilized microbial systems Curr Opin Biotechnol 2012 10.1016/j.copbio.2012.05.001 22651991

[B6] Posfai G Plunkett G Feher T Frisch D Keil GM Umenhoffer K Kolisnychenko V Stahl B Sharma SS de Arruda M Emergent properties of reduced-genome Escherichia coli Science 2006 312 5776 1044 1046 10.1126/science.1126439 16645050

[B7] Leprince A de Lorenzo V Voller P van Passel MW Martins Dos Santos VA Random and cyclical deletion of large DNA segments in the genome of Pseudomonas putida Environ Microbiol 2012 14 6 1444 1453 10.1111/j.1462-2920.2012.02730.x 22429517 PMC3429869

[B8] Constante M Grunberg R Isalan M A biobrick library for cloning custom eukaryotic plasmids PLoS One 2011 6 8 e23685 10.1371/journal.pone.0023685 21901127 PMC3161993

[B9] Smolke CD Building outside of the box: iGEM and the BioBricks Foundation Nat Biotechnol 2009 27 12 1099 1102 10.1038/nbt1209-1099 20010584

[B10] Hesselman MC Odoni DI Ryback BM de Groot S van Heck RG Keijsers J Kolkman P Nieuwenhuijse D van Nuland YM Sebus E A multi-platform flow device for microbial (co-) cultivation and microscopic analysis PLoS One 2012 7 5 e36982 10.1371/journal.pone.0036982 22606321 PMC3351485

[B11] Boyle PM Burrill DR Inniss MC Agapakis CM Deardon A Dewerd JG Gedeon MA Quinn JY Paull ML Raman AM A BioBrick compatible strategy for genetic modification of plants J Biol Eng 2012 6 1 8 10.1186/1754-1611-6-8 22716313 PMC3537565

[B12] Kelly JR Rubin AJ Davis JH Ajo-Franklin CM Cumbers J Czar MJ de Mora K Glieberman AL Monie DD Endy D Measuring the activity of BioBrick promoters using an in vivo reference standard J Biol Eng 2009 3 4 10.1186/1754-1611-3-4 19298678 PMC2683166

